# The effect of *Lactobacillus brevis* KB290 against irritable bowel syndrome: a placebo-controlled double-blind crossover trial

**DOI:** 10.1186/1751-0759-6-16

**Published:** 2012-08-03

**Authors:** Katsumi Murakami, Chizu Habukawa, Yukihiro Nobuta, Naohiko Moriguchi, Tsukasa Takemura

**Affiliations:** 1 Department of Pediatrics, Kinki University Sakai Hospital, 2-7-1 Harayamadai, Minami-ku, Sakai, Osaka 590-0132, Japan; 2 Department of Pediatrics, Minami Wakayama Medical Center, 27-1 Takinai-machi, Tanabe-shi, Wakayama 646-0015, Japan; 3 Probiotics Research Group, Nature-Wellness research Department, Research Institute, KAGOME Co., LTD. 17 Nishitomiyama, Nasushiobara, Tochigi 329-2762, Japan; 4 Department of Pediatrics, Kinki University Faculty of Medicine, 377-2 ,Onohigashi, Osakasayama-shi, Osaka 589-8511, Japan

**Keywords:** Irritable bowel syndrome, *Lactobacillus brevis* KB290, Probiotic

## Abstract

**Background:**

Irritable bowel syndrome (IBS) is a functional disorder of the digestive tract that causes chronic abdominal symptoms. We evaluated the effects of *Lactobacillus brevis* KB290 (KB290), which has been demonstrated to be effective at improving bowel movements and the composition of intestinal microflora, on IBS symptoms.

**Methods:**

We performed a placebo control double-blind cross matched trial. Thirty-five males and females (aged 6 years and above) who had been diagnosed with IBS according to the Rome III criteria were divided into 2 groups, and after a 4-week pre-trial observation period, they were administered test capsules containing KB290 or placebo for 4 weeks (consumption period I). Then, the capsule administration was suspended for 4 weeks in both groups (washout period), before the opposite capsules were administered for a further 4 weeks (consumption period II). Fecal samples were collected on the first day of the pre-consumption observation period, the last day of consumption period I, the last day of the washout period, and the last day of consumption period II. In addition, the subjects’ IBS symptoms and quality of life (QOL) and any adverse events that they experienced were evaluated.

**Results:**

No significant difference in IBS symptoms was noted among the various periods. However, the mean QOL scores were improved during the test capsule consumption.

The frequencies of watery and mushy feces were significantly lower in the test capsule consumption period than during the pre-consumption observation period, and the frequency of abdominal pain was significantly reduced in the test capsule consumption period compared with the other periods.

The frequency of the genus *Bifidobacterium* was significantly higher, and that of the genus *Clostridium* was significantly lower, after the test capsule consumption than after the placebo consumption. The frequencies of the genera *Lactobacillus*, *Bacteroides*, and *Enterococcus* were also investigated, but no differences in their frequencies were detected between the placebo and test capsule consumption periods.

**Conclusions:**

Probiotics, the safety of which has been established, are used widely in various foods and can now be purchased readily. The results of the present study suggest that KB290 is useful for early intervention in IBS.

## Background

Irritable bowel syndrome (IBS) is a functional disorder of the digestive tract that causes chronic symptoms such as abdominal pain and discomfort and abnormal bowel movements such as diarrhea and constipation but exhibits no organic abnormalities [1]. Its etiology and pathology are yet to be elucidated. However, abnormalities in brain-gut interactions; i.e., excessive stimulation of the digestive tract by the central nervous system during stress; the disturbance of sensory and cognitive mechanisms by disorders of the digestive tract, infection, or inflammation of the digestive tract; and changes in the intestinal microflora have been reported to be involved [2]. This suggests that the symptoms of IBS can be alleviated by restoring the normal intestinal flora and that we need to obtain a greater understanding of intestinal microflora [3]. Analyses of the microflora in IBS patients and healthy individuals have indicated that differences exist between them [4], but no intestinal bacteria that directly trigger the condition have been identified.

Some clinical trials have indicated that probiotics are effective at alleviating IBS symptoms and improving the quality of life (QOL) of IBS patients [5-8]. Also, the mechanism responsible for the beneficial effects of probiotics has not been clarified although improvements in the composition of the intestinal flora and the suppression of intestinal inflammatory reactions have been proposed as possible mechanisms [7,9].

Therefore, in this study we evaluated the effects of *Lactobacillus brevis* KB290 (KB290), which is a plant-derived strain of lactic acid bacillus isolated from *suguki* (a traditional pickle produced in Kyoto) that has been demonstrated to be effective at improving bowel movements and the composition of the intestinal microflora [10-13], on IBS symptoms and examined its effects on intestinal microflora to clarify the mechanism by which it induces the abovementioned improvements.

## Methods

### Study design

**Figure 1:**
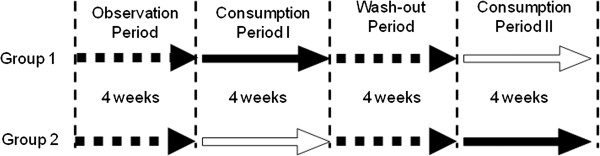
**Study design.** The subjects were divided into 2 groups, and after a 4-week pre-trial observation period, administered test capsules containing KB290 or placebo capsules not containing KB290 at 1 capsule/day for 4 weeks (consumption period I). Then, the capsule administration was suspended for 4 weeks in both groups (washout period), and the capsules opposite to those administered first were administered for 4 weeks (consumption period II).

The patients were randomly divided into 2 groups, which were matched in age and gender, according to a computer generated randomized list. After a 4-week pre-trial observation period, they were administered test capsules containing KB290 (freeze-dried KB290 bodies at a dose of ≥1.0 × 1010 cfu/capsule mixed with corn starch, maltitol syrup, hydroxypropyl methylcellulose, and calcium stearate) or placebo capsules that did not contain KB290 at a rate of 1 capsule/day for 4 weeks (consumption period I). For the next 4 weeks, both groups stopped consuming the capsules (washout period). After the washout period, the groups were administered the opposite capsules to those that they had taken during the 1st consumption period for a further 4 weeks (consumption period II). The study protocol was approved by the ethics committees of the participating institutions (Figure 1).

### Determination of the viable KB290 cell count

Counts of viable KB290 cells were obtained during the preparation of the test capsules and after the end of their administration. Then, after carefully weighing out 1.0 g of the powder obtained from 5 capsules, it was serially diluted using 0.1% (w/v) agar dissolved in physiological saline, and a 50 μL suspension was applied to 15 mL plate count agar (PCA) containing bromocresol purple (BCP) (Eiken Chemical). The suspension was cultured at 35°C for 72 hours, and then the number of colonies was counted. Since each capsule contained 0.34 - 0.38 g KB290 (standard content, 0.36 g), the number of living KB290 cells was calculated by assuming the KB290 content per capsule to be 0.34 g.

### Patients

Thirty-five males and females (aged 6 years and above) who had been diagnosed with IBS according to the Rome III criteria [14] were selected. We calculated that in order for our model to possess a power of 80% to detect a difference between the two consumption periods when α = 0.05, we would need to analyze 20 patients. In addition, we assumed a worst-case control rate of 67%. Accordingly, we recruited 35 patients for this study. To ensure the safety of the patients and eliminate the effects of factors other than the test capsule on the study outcomes, any patients who had serious disorders other than IBS or allergies to a material contained in the test capsule, had undergone gastrointestinal tract surgery other than appendectomy, had participated in another clinical study within the 3 months prior to the beginning of this study, or who had an infectious disease were excluded. During the patient recruitment, the intentions of the study and its procedures were thoroughly explained to the participants, and written consent to participate in the study was obtained in compliance with the Helsinki Declaration. During the study, the patients were instructed not to eat greater than usual amounts of foods that contained lactic acid bacilli (other than the test capsule).

Any subjects who had been administered antibiotics during the study period, had consumed foods that are known to markedly affect the condition of the bowel, that did not consume the test capsule as instructed, or whose diary entries were defective were excluded from the analysis.

### Observation of IBS symptoms

The subjects (or their parents if the subjects could not keep a diary by themselves) kept a daily bowel movements diary during the study. The recorded items were the frequency of bowel movements, fecal properties (amount, color, shape), physical sensations experienced after bowel movement, time required for bowel movements, abdominal symptoms (frequency of attacks of abdominal pain), compliance with the administration schedule, and the consumption of foods that affect the condition of the bowel. Regarding the frequency of bowel movements, the mean weekly number of bowel movements was calculated separately for the pre-consumption observation period, washout period, placebo consumption period, and test capsule consumption period. The volume of feces was recorded in terms of the number of chicken eggs, and the mean value per bowel movement was calculated for each period. Concerning the shape and color of feces and the physical sensations experienced after bowel movement, mean scores were calculated for each period. As for the time required for bowel movements, the mean time of one bowel movement was calculated for each period. The frequency of attacks of abdominal pain was expressed as the mean weekly number for each period.

### Analysis of fecal microflora

Fecal samples were collected on the first day of the pre-consumption observation period, the last day of consumption period I, the last day of the washout period, and the last day of consumption period II. Immediately after its discharge, a sample of the targeted feces was suspended in a vessel containing a DNA preservation medium (GCT buffer), the suspension was stored at 4°C until analysis, and the intestinal microflora it contained were analyzed using the terminal restriction fragment length polymorphism (T-RFLP) method [15]. After 16S rRNA had been extracted from the fecal suspension using the Magtration System 12GC automatic nucleic acid extraction machine (Precision System Science), it was reverse transcribed using the universal primer 516F-1510R (Life Technologies Japan) and digested using the restriction enzyme *Bsl*I (New England Biolabs). The digested cDNA fragments were subjected to capillary electrophoresis using an ABI3100 genetic analyzer (Applied Biosystems), the peak pattern of the DNA fragments obtained by absorptiometry at 260 nm was compared with that of the DNA fragments of the standard, and the bacterial species present in the feces were determined. The percentage areas of the peaks for the genera *Bifidobacterium* and *Clostridium* relative to the total area of all detected peaks were calculated, and the percentage changes in the areas of these peaks were also calculated to analyze the changes induced in the composition of the intestinal microflora by the test food or placebo.

### Evaluation of QOL

QOL was evaluated before and after the consumption of the test capsule by assessing the physical activity, emotional problems, social activity, pain, and overall health state of the subjects using a 5-point scale and the Japanese version of the Dartmouth COOP charts [16], and mean scores were calculated for each period.

### Evaluation of adverse events

The adverse events that occurred during the study were evaluated on the basis of the subjects’ bowel movement diaries and interviews conducted at the end of the study. Abdominal pain and diarrhea, and adverse events that might have been caused by the consumption of the test capsule were recorded, and their frequencies during the test capsule consumption period were compared with those during the other periods.

### Statistical analyses

The results for each evaluated item were totaled for each subject for the pre-consumption observation period, washout period, placebo consumption period, and test capsule consumption period and statistically analyzed. The frequency of abdominal pain, frequency of bowel movements, fecal properties (volume, shape, color, smell), physical sensations experienced after bowel movement, time required for bowel movements, and frequencies of the genera *Bifidobacterium* and *Clostridium* among the fecal microflora were examined using the paired *t*-test with Bonferroni’s correction. The significance of the differences in the QOL score was examined using the Steel-Dwass method. Statistical analyses were performed using the SPSS software (ver15.0, IBM) at a significance level of *p*<0.05.

## Results

### Number of living KB290 cells

The mean number of living KB290 bacteria in the test capsule was 1.2 ± 0.3×10 [10] cfu/capsule on the day of preparation (December 8, 2008) and 5.5 ± 0.4× 10 [9] and 6.8 ± 0.3×10 [9] cfu/capsule on the last day of test capsule consumption in the first (May 25, 2009) and last (September 5, 2009) subjects, respectively. The mean of these 3 measurements was 8.1 ± 0.4×10 [9] cfu/capsule. Also, no KB290 bacteria were detected in the placebo capsule at any point.

### Analysis group

**Figure 2:**
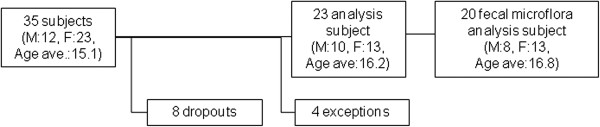
**Analysis group.** Thirty-five persons who volunteered to participate in the study were interviewed and all were enrolled, because none matched any of the exclusion criteria. Eight of them dropped out during the study, and, from those who completed the study, 23 (10 males and 13 females, mean age 16.2 ± 10.5 years) were selected as an analysis group by excluding 2 with defects in diary entries and 2 in whom the number of days with test capsule consumption was insufficient according to a premeditated procedure. The fecal microflora was analyzed in 20 subjects of the analysis group in whom data could be obtained in all periods.

Thirty-five patients were interviewed and enrolled. Eight of them dropped out during the study, and of those who completed the study, four were excluded because of problems with their diary entries or with their compliance with the test or placebo capsule consumption protocol, thus 23 patients (10 males and 13 females, mean age: 16.2 ± 10.5 years) were included in the analysis (Figure 2). The fecal microflora of 20 subjects for whom data were obtained in all periods were analyzed. Three patients were excluded from the fecal microflora analysis because of problems with the fecal collection. No significant differences were noted in any of the examined items between Groups 1 and 2.

### IBS symptoms

Table 1shows our findings regarding the frequency of bowel movements, the volume and color of the subjects’ feces, the physical sensations they experienced after bowel movements, and the time they required for bowel movements. No significant difference was noted in any of these items among the various periods.

**Table 1:** IBS symptoms

	**Observation period**	**Wash-out period**	**Consumption period**	
			**Placebo**	**KB290**
Frequency of bowel movement (times/week)	8.8 ± 3.5	7.8 ± 2.5	7.0 ± 3.8	8.0 ± 3.1
Fecal volume (hen’s egg-size)/time	2.0 ± 1.5	1.7 ± 1.1	1.8 ± 1.5	2.1 ± 1.8
Fecal color (score mean value)	3.6 ± 1.5	3.8 ± 1.4	3.6 ± 1.4	3.5 ± 1.8
Feeling of bowel movement (score mean value)	3.2 ± 1.2	3.6 ± 0.8	3.0 ± 1.5	3.8 ± 1.3
Amount of time for bowel movement (minutes/time)	5.5 ± 4.8	5.9 ± 5.1	5.2 ± 5.0	5.3 ± 4.3

Values are expressed as Means ± SD, n = 23.

**Figure 3:**
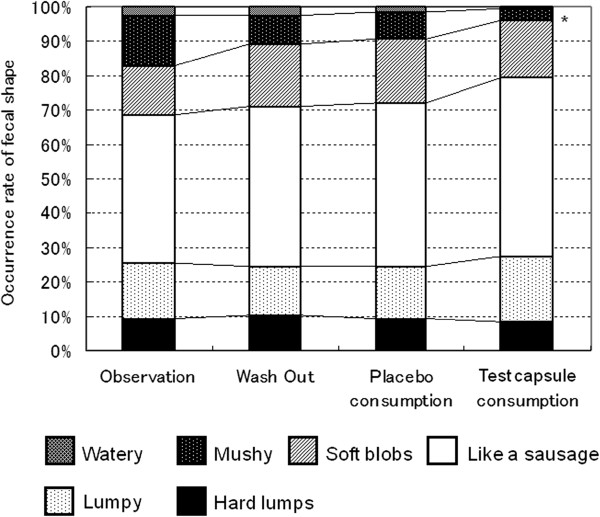
**The frequencies of the appearance of various fecal shapes in each period.** In the placebo consumption period, no significant difference was noted compared with the washout period, but the frequencies of watery and mushy feces were significantly lower in the test capsule consumption period compared with the pre-consumption observation period.

Figure 3 shows the frequencies of various fecal shapes in each period. No significant difference in the frequency of any fecal shape was detected between the placebo consumption period and the washout period; however, the frequencies of watery and mushy feces were significantly lower in the test capsule consumption period than in the pre-consumption observation period.

**Figure 4:**
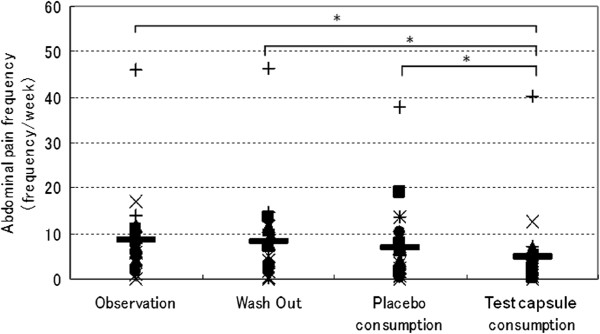
**The frequency of abdominal pain in each period.** The frequency of abdominal pain was significantly reduced in the test capsule consumption period compared with the other periods.

Figure 4 shows the frequency of abdominal pain in each period. The frequency of abdominal pain was significantly reduced in the test capsule consumption period compared with the other periods.

### Intestinal microflora

**Figure 5:**
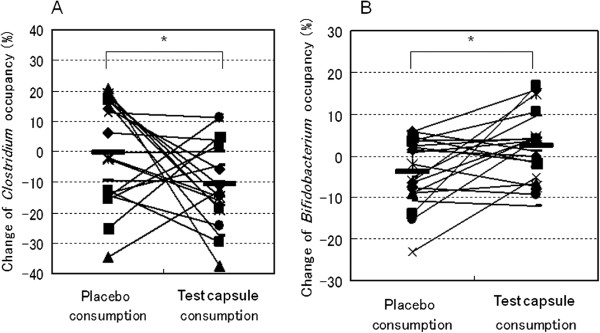
** Intestinal microflora.** Changes in the percentages of the genera Bifidobacterium and Clostridium were compared by T-RFLP analysis. As a result, the percentage of the genus Bifidobacterium was significantly higher, and that of the genus Clostridium was significantly lower, after test capsule consumption than after placeb consumption.

The frequencies of the genera *Bifidobacterium* and *Clostridium* were assessed by T-RFLP analysis. As a result, we found that the frequency of the genus *Bifidobacterium* was significantly higher, and that of the genus *Clostridium* was significantly lower, after the test capsule consumption than after the placebo consumption (Figure 5). The frequencies of the genera *Lactobacillus (placebo : KB290 = 4.1% : 7.2%)*, *Bacteroides (placebo : KB290 = 33.8% : 34.3%)*, and *Enterobacter (placebo : KB290 = 8.4% : 5.8%)* were also investigated, but no significant differences in their frequencies were detected between the placebo and test capsule consumption periods.

### Evaluation of QOL

The Japanese version of the Dartmouth COOP Chart indicates that the lower the score for a QOL item, the fewer problems there are with that item. Table 2 shows the scores for physical activity, emotional problems, social activity, pain, and overall health state. While no significant difference was noted in any item among the periods, the mean score for all items was reduced in the test capsule consumption period.

**Table 2:** The scores for physical activity

	**Observation Period**	**Wash-out period**	**Consumption Period**	
			**Placebo**	**KB290**
Physical	2.3 ± 0.9	2.5 ± 0.9	2.1 ± 0.9	2.0 ± 0.6
Emotional	2.5 ± 0.7	2.6 ± 0.9	2.5 ± 0.9	2.4 ± 0.7
Social Activities	2.3 ± 0.7	2.4 ± 0.8	2.1 ± 0.7	2.1 ± 0.7
Pain	2.8 ± 0.9	2.8 ± 1.1	2.6 ± 1.0	2.5 ± 0.9
Overall Health	3.0 ± 0.8	3.0 ± 0.8	2.9 ± 0.8	2.8 ± 0.8

Values are expressed as Means ± SD, n = 23.

### Adverse events

According to the subjects’ bowel movement diary entries and interview responses, no adverse events that were suspected to be related to the test capsule were observed during the study other than IBS symptoms, and no increase in the frequency of IBS symptoms such as abdominal pain or diarrhea was noted.

## Discussion

We evaluated the effects of KB290 as a probiotic on IBS symptoms and intestinal microflora in IBS patient. As a result, we found that taking KB290 improved the QOL, significantly alleviated their abdominal pain and improved their fecal properties, and significantly changed the composition of their intestinal microflora.

Previous reports have suggested that some probiotics alleviate the symptoms of IBS patients and improve their QOL and the composition of their intestinal microflora [5-8]. The genera *Bifidobacterium* and *Lactobacillus,* and *Enterobacter* and *Clostridium,* have been reported to display lower and higher frequencies, respectively, among the microflora of IBS patients than among those of healthy individuals [17]. In the present study, we focused our analysis on the frequencies of the genera *Bifidobacterium* and *Clostridium*, which have been suggested to be related to IBS symptoms. Previous studies have shown that in healthy individuals with a tendency for constipation the frequencies of the genera *Bifidobacterium* and *Lactobacillus* increase and the frequency of the genus *Enterobacter* decreases after the consumption of KB290. In this study, not only did the frequency of the genus *Bifidobacterium* increase after KB290 intake, but that of the genus *Clostridium* also decreased. However, no increase in the frequency of the genus *Lactobacillus* or decrease in that of the genus *Enterococcus* was detected, probably because of the large inter-individual variations in human intestinal microflora. Human intestinal microflora have been shown to change with age and diet [18-20]. In this study, no dietary restrictions were imposed on the subjects, and this might have been related to the lack of change in the frequencies of these genera after the test capsule consumption.

It is very difficult to obtain a detailed overview of intestinal microflora. For example, studies of intestinal microflora composition almost exclusively rely on the quantitative culturing of microbes from fecal samples. However, recent nucleic acid studies have indicated that the majority of bacteria in a variety of ecosystems are different from those detected in culture [21]. Therefore, only part of the intestinal microflora of IBS patients can be examined by conventional techniques, and abnormalities in intestinal microbes that cause IBS might have been overlooked. Recently, the utility of molecular biological techniques such as RT-PCR analysis, denaturing gradient gel electrophoresis (DGGE), FISH, T-RFLP, and pyrosequencing for analyzing the 98% of intestinal microflora that have so far escaped analysis was evaluated, but no intestinal bacterium that can directly cause IBS has been identified [16,22-25]. In this study, the intestinal microflora were analyzed using the T-RFLP technique, a molecular biological method, but our analysis primarily focused on the genera *Bifidobacterium* and *Clostridium*. In the future, as analytical methods for intestinal microflora are developed, yielding a larger number of reports, intestinal bacterial species that are closely involved in the initiation and exacerbation of IBS might be identified.

A recent study suggested that the intestinal microflora plays an important role in brain-gut interactions [26].Another report indicated that intestinal microflora affect the central nervous system [27], and changes in host behavior associated with changes in intestinal microflora have been observed in animal experiments [28]. Thus, based on recent findings regarding the functions of microbiota, the “microbiota - gut - brain axis” has been suggested as a new concept that describes the functional relationship that exists among microbiota, the gut, and the brain [29].

It was reported that intestinal inflammation is involved in the pathogenesis of IBS and that probiotics alleviate the symptoms of IBS by normalizing the balance of anti-inflammatory and proinflammatory cytokines. However, we could not determine whether KB290 affects cytokine expression in this study [30].

In this study, KB290 was shown to alleviate IBS symptoms and affect the frequencies of the genera *Bifidobacterium* and *Clostridium* among the constituents of the intestinal microflora, but the mechanism responsible for this could not be clarified. In the future, it will be necessary to more closely analyze the alterations in biomarker levels associated with the changes in intestinal microflora composition and symptom alleviation induced by KB290 intake and to clarify the changes KB290 causes in the composition of the intestinal microflora in greater detail. However, in consideration of the recently clarified role of intestinal microflora in the mechanisms of IBS symptom alleviation and the etiology of the disease, the intake of KB290, a probiotic, is considered to be able to contribute to symptomatic improvement in such patients.

## Conclusions

Probiotics, the safety of which has been established, are used widely in various foods and can now be purchased readily. IBS has been reported to occur in children as young as 6, and its symptoms, such as abdominal pain, abdominal distension, and diarrhea, impair the QOL of IBS patients. In addition, as it takes a long time to cure IBS, early therapeutic intervention is important. The results of the present study suggest that KB290 is useful for early intervention in such patients.

## Competing interests

All authors have no conflicts of interest to disclose.

## Authors' contributions

KM, CH, YN designed the study and conception. KM, CH, NM, TT collected data. KM, YN analysed and interpreted the data. KM, CH prepared the manuscript, with input from KM and CH additionally all authors must approve the final version of the manuscript.
